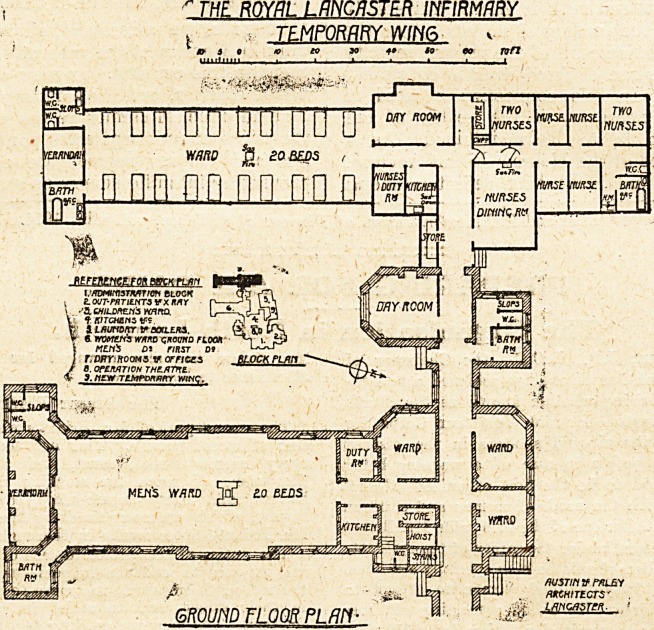# The Royal Lancaster Infirmary (Temporary Wing)

**Published:** 1918-01-26

**Authors:** 


### HOSPITAL ARCHITECTURE AND CONSTRUCTION.

### THE ROYAL LANCASTER INFIRMARY (Temporary Wing).

THIS temporary addition to the infirmary has been erected to provide accommodation for munition workers, a large number of whom have come to Lancaster for this work.

The wing has been erected on the west side of the existing building, and is one storey high. It contains a ward for twenty beds, with a bath-room and sanitary offices arranged in two projecting wings at the south end, and a verandah between. These offices are approached direct from the ward without the usual "cut-off" lobby. The w.c.s and bed-pan sink are in consequence unpleasantly near to the end bed. It would have been a great improvement if the wings had been connected to the ward by lobbies placed diagonally, as by this plan the verandah would have been considerably increased.

At the north end of the ward is a large day-room, with a duty-room and ward kitchen. The word "duty-room" is usually used as meaning "ward kitchen"; in this case we presume it means probably sisters' room. On the other side of the corridor is accommodation for nurses, comprising a dining-room, two double bedrooms, and four single bedrooms, with a bathroom, w.c., and housemaids' closet.

The block is built with brick foundations up to the floor level, timber framing above, lined on the inside with fibrous plaster slabs and on the outside with asbestos cement slabs; the whole block standing on a layer of concrete. The heating is by hot water at low pressure.

The cost of the building was about £1,900, which is at the rate of £95 per bed—a considerable sum for a building intended for temporary use. The architects were Messrs. Austin and Paley, of Lancaster.

## Figures and Tables

**Figure f1:**